# Outcome prediction in aneurysmal subarachnoid hemorrhage: a comparison of machine learning methods and established clinico-radiological scores

**DOI:** 10.1007/s10143-020-01453-6

**Published:** 2021-01-20

**Authors:** Nora Franziska Dengler, Vince Istvan Madai, Meike Unteroberdörster, Esra Zihni, Sophie Charlotte Brune, Adam Hilbert, Michelle Livne, Stefan Wolf, Peter Vajkoczy, Dietmar Frey

**Affiliations:** 1 Department of Neurosurgery, Charité Universitaetsmedizin Berlin, Charitéplatz 1, 10117 Berlin, Germany; 2 CLAIM – Charité Lab for AI in Medicine, Charité Universitaetsmedizin Berlin, Charitéplatz 1, 10117 Berlin, Germany; 3 School of Computing and Digital Technology, Faculty of Computing, Engineering and the Built Environment, Birmingham City University, 15 Bartholomew Row, Birmingham, B5 5JU UK; 4 Technological University Dublin, Aungier St, Dublin, D02 HW71 Ireland

**Keywords:** Aneurysmal subarachnoid hemorrhage, Outcome prediction, Deep learning, Artificial neural net, Tree boosting

## Abstract

Reliable prediction of outcomes of aneurysmal subarachnoid hemorrhage (aSAH) based on factors available at patient admission may support responsible allocation of resources as well as treatment decisions. Radiographic and clinical scoring systems may help clinicians estimate disease severity, but their predictive value is limited, especially in devising treatment strategies. In this study, we aimed to examine whether a machine learning (ML) approach using variables available on admission may improve outcome prediction in aSAH compared to established scoring systems. Combined clinical and radiographic features as well as standard scores (Hunt & Hess, WFNS, BNI, Fisher, and VASOGRADE) available on patient admission were analyzed using a consecutive single-center database of patients that presented with aSAH (*n* = 388). Different ML models (seven algorithms including three types of traditional generalized linear models, as well as a tree bosting algorithm, a support vector machine classifier (SVMC), a Naive Bayes (NB) classifier, and a multilayer perceptron (MLP) artificial neural net) were trained for single features, scores, and combined features with a random split into training and test sets (4:1 ratio), ten-fold cross-validation, and 50 shuffles. For combined features, feature importance was calculated. There was no difference in performance between traditional and other ML applications using traditional clinico-radiographic features. Also, no relevant difference was identified between a combined set of clinico-radiological features available on admission (highest AUC 0.78, tree boosting) and the best performing clinical score GCS (highest AUC 0.76, tree boosting). GCS and age were the most important variables for the feature combination. In this cohort of patients with aSAH, the performance of functional outcome prediction by machine learning techniques was comparable to traditional methods and established clinical scores. Future work is necessary to examine input variables other than traditional clinico-radiographic features and to evaluate whether a higher performance for outcome prediction in aSAH can be achieved.

**Supplementary Information:**

The online version contains supplementary material available at 10.1007/s10143-020-01453-6.

## Introduction

Scoring systems help clinicians to classify the severity of a disease, to estimate the natural course, and to select treatment strategies[1, 23]. For aneurysmal subarachnoid hemorrhage (aSAH), the Hunt and Hess scale and the WFNS scale have been used in clinical routine for many decades. Both scores are based on clinical patient characteristics in terms of consciousness and neurological deficits [15, 28]. Numerous radiographic scores were introduced, using qualitative imaging features like the dispersion of the subarachnoid blood clot as well as the presence of intraventricular hemorrhage (IVH) or intracerebral hemorrhage (ICH)[13, 14]. The first semi-quantitative radiological predictive tool was proposed by the Barrow Neurological Institute (BNI) in 2012 [35]. However, to date, neither clinical nor radiographic scores reached the accuracy needed for definite decision-making [9, 34]. Combinations of radiographic and clinical features using traditional statistic methods have also not resulted in improved predictions [6, 17].

There is a clinical need to find tools that facilitate individualized risk stratification at an early time point of the disease to responsibly allocate resources (e.g., intensive care unit (ICU) beds) and decide on treatment strategies. Recently, machine learning (ML) approaches are increasingly applied in healthcare. Such techniques include support vector machines, decision trees, Bayesian approaches, and artificial neural networks. They may improve the clinical performance of predictive models [27, 30]. In this context, especially artificial neural nets (ANN) and methods of tree boosting, a decision tree–based algorithm, showed better performance than traditional ML approaches such as linear and logistic regression for numerous applications [12, 18, 37]. However, the substantial heterogeneity of clinical questions, input and output variables, and applied algorithms may reduce traceability and reproducibility [24, 32].

We aimed to examine in this study whether applying ML techniques improves the performance of outcome prediction in aSAH. First, we analyzed whether existing scores would benefit from the application of ML techniques. Second, we combined a set of traditional clinico-radiological features that showed to be relevant for patient outcome with availability on admission and compared its predictive performance to traditional clinical scores to maintain transparency and comparability with existing studies.

## Materials and methods

### Data collection

We included radiographic and clinical data of consecutive patients after aSAH treated at two hospitals of a single academic institution between 2009 and 2015. The study was approved by the ethics review board of Charité Universitaetsmedizin Berlin (EA1/291/14). Patients with documented aSAH on CT or positive lumbar puncture were enrolled in the study. Patients with bleeding sources other than an intracranial aneurysm documented by CT angiogram or digital subtraction angiography were excluded. Clinical scores were applied on admission and radiographic scores were calculated based on admission CT.

### Patient management

The local treatment protocol was previously described [8, 25]. In brief, patients were treated according to international guidelines with early aneurysm occlusion, clinical and/or multimodal invasive neuromonitoring in the ICU [2].

### Outcome assessment

The primary outcome measure in our study was functional outcome using the Modified Rankin Scale (mRS) [31]. Clinical outcome was acquired from files during scheduled control visits 6–12 months after the initial hemorrhage. If sufficient information was not available for mRS determination, a systematic telephone interview was conducted. Both assessments were blinded to initial SAH severity grading. Outcome was dichotomized as favorable (mRS 0–2) or unfavorable (mRS 3–6).

### Scores

CT, clinical, and combined scores were applied according to the respective literature [13–15, 28, 35]. A routine assessment of Hunt and Hess grading, neurological deficits, and GCS was performed prospectively on admission and electronically documented. Calculation of WFNS score was therefore indirectly possible based on GCS. Radiographic data were retrospectively assessed by an experienced neurosurgeon blinded for outcome. VASOGRADE was calculated based on this retrospective and prospective data assessment and according to previous literature [3]. Moreover, clinical data assessment included patient age, sex, and pupillary state (equal, reactive to light vs. fixation of one or more pupil). Additional radiographic features that were included were presence of ICH, IVH, subdural hematoma (SDH), and midline shift (MLS) larger than 5 mm. Aneurysm size and aneurysm position dichotomized for posterior or anterior location were assessed with the help of CT angiography and/or digital subtraction angiogram (DSA) on admission. An overview of the scores used for prediction is presented in Table 1.

**Table 1:** Overview of the 8 different feature selections. ICH = intracranial hemorrhage; IVH = intraventricular hemorrhage; SDH = subdural hemorrhage; GCS = Glasgow Coma Scale score; BNI = Barrow Neurological Institute scale; WFNS = World Federation of Neurosurgical Societies

	Feature(s)
1	Hunt and Hess Score value
2	WFNS score value
3	Original Fisher score value
4	Modified Fisher score value
5	VASOGRADE score value
6	BNI score value
7	Glasgow Coma Scale value
8	Age, GCS score, sex, pupil status, presence of IVH, presence of ICH, presence of midline shift > 5 mm, presence of SDH, localization anterior circulation or other, BNI score

### Feature selection

The available database consisted of 408 patients. Of these, 20 patients did not have mRS values and were thus excluded resulting in the final number of 388 patients. There were very few missing values present (age 0.8%, ICH 0.3%, MLS 0.5%, SDH 0.3%, localization 0.8%, VASOGRADE score 1%). We used mean/mode imputation in each fold to impute missing values (see section “Model training and validation”).

For input features, inclusion criteria were a ratio of at least 1 to 4 for binary variables (absence/presence) and no more than 10% missing values. As an exception, we included pupil status (13.4 % of patients with pathological pupil status) due to its clinical importance (20). The following features were available: age, sex, pupil status, presence of IVH, presence of ICH, presence of MLS, presence of SDH, and the localization of the aneurysm. Categorical features with more than two or more categories were transformed into binary features as they had too few instances per category. Pupil status was dichotomized to “both pupils reactive to light” vs “pathological.” Radiologically defined ICH was dichotomized to “yes”/”no.” Radiologically defined change in the brain midline was dichotomized to shift > 5 mm “yes”/”no.” Location of the aneurysm was dichotomized to anterior circulation “yes”/”no.” Thus, all resulting features were either binary categories or continuous.

### Model selection

We trained a model for each single score (HH, WFNS, original Fisher, modified Fisher, VASOGRADE combined, BNI, and GCS). Additionally, we a priori constructed a combined feature set of selected scores (GCS, BNI) and individual features in a way that all clinically relevant (age, pupil state, and GCS) and radiographically important parameters (including IVH, ICH, SDH, MLS, and BNI for semi-quantitative description of the thickness of subarachnoidal blood) available on admission were included. The final set of input features for each tested model is listed in Table 1.

### Machine learning framework

The ML framework was written in Python using standard ML libraries. The main framework has previously been described in full technical detail in an open access publication [38]. The current framework code is available on GitHub (https://github.com/prediction2020/explainable-predictive-models). In a supervised ML approach, the above-mentioned clinical parameters and clinical scores (see also Table 1) were used to predict the final outcome of aSAH patients according to mRS. The applied dichotomization resulted in 181 positive (favorable outcome) and 207 negative (unfavorable outcome) cases. This small imbalance causes negligible bias and therefore did not warrant a sub-sampling approach limiting the available data for model training.

### Applied algorithms

Seven different algorithms were applied for all eight feature selections. We used three types of generalized linear models (GLM): a plain GLM, an L1 regularized GLM (equivalent to Lasso logistic regression), and a GLM elastic net adding an additional L2 regularization. Additionally, the CatBoost tree boosting algorithm, a support vector machine classifier (SVMC), a Naive Bayes (NB) classifier, and a multilayer perceptron (MLP) artificial neural net were used. For feature selection 8 (the only model with more than one feature, see also Table 1), feature importance ratings were calculated, for all seven algorithms, using SHapley Additive exPlanations (SHAP) values. A full technical overview of the algorithms and the feature importance calculations are available in the open access publication of the applied framework [38] and the GitHub page of our framework (see above). Since multicollinearity may confound the predictive performance, we estimated multicollinearity of the features using the variance inflation factor (VIF) [22].

### Model training and validation

The data were randomly split into training and test sets with a corresponding 4:1 ratio. Mean/mode imputation and feature scaling using zero-mean unit variance normalization based on the training set was performed on both sets. The models were then tuned using 10-fold cross-validation. The whole process was repeated in 50 shuffles.

### Performance assessment

The model performance was tested on the test set using receiver operating characteristic (ROC)-analysis by measuring the area under the curve (AUC) as the primary measure. Additional performance measures were accuracy, average class accuracy, precision, recall, f1 score, negative predictive value, and specificity. To estimate calibration of the models, the Brier score was calculated. All measures are given as the median over 50 shuffles.

### Interpretability assessment

The absolute values of the calculated feature importance scores were scaled to unit norm to provide comparable feature rating across models: for each of the 50 shuffles, the calculated importance scores were rescaled to the range [0,1] with their sum equal to one. Then, for each feature, the mean and standard deviation over the shuffles were calculated and reported as the final rating measures.

## Results

### Patient characteristics and importance of features

Three hundred eighty-eight patients with a median age of 54 years (IQR 46; 63) and a female:male ratio of 2.3 were included in the final analysis. Clinical and radiographic patient characteristics are depicted in Table 2. Functional outcome was evaluated after a median of 10 months (IQR 6; 17).

**Table 2:** Clinical, radiographic, and treatment characteristics of patients with aSAH. Pathological pupil reaction describes a pupil reaction other than pupils equal and reactive to light. *WFNS* World Federation of Neurological Societies, *IVH* intraventricular hemorrhage, *ICH* intracerebral hemorrhage, *SDH* subdural hemorrhage, *SAH* subarachnoidal hemorrhage, *ACA* anterior cerebral artery, *MCA* middle cerebral artery, *ICA* internal cerebral artery, *mRS* Modified Rankin Scale. Localization of the aneurysm was available for 380/388 patients

			% (*n*)
Clinical features	Pathological pupil reaction		13.4% (52)
	GCS at admission	3	32.3% (125)
		4–8	8.7% (34)
		9–12	9.0% (35)
		13–15	50.0% (194)
Clinical scores	WFNS	I	36.6% (142)
		II	9.8% (38)
		III	3.4% (13)
		IV	12.6% (49)
		V	37.6% (146)
	Hunt and Hess	I	24.2% (94)
		II	17.5% (68)
		III	14.7% (57)
		IV	14.2% (55)
		V	29.4% (114)
Radiographic features	IVH		44.3% (172)
	ICH		32.0% (124)
	SDH		6.5% (25)
	Midline shift (> = 5 mm)		23.1% (89)
	Thickness of SAH (BNI)	< 5 mm (1°)	6.4% (25)
		6–10 mm (2°)	16.0% (62)
		11–15 mm (3°)	29.9% (116)
		15–20 mm (4°)	32.0% (124)
		> 25 mm (5°)	15.7% (61)
	Aneurysm location	ACA	35.8% (136)
		ICA	19.2% (73)
		MCA	26.1% (99)
		Posterior circulation	18.9% (72)
Radiographic scores	Modified Fisher	0	4.6% (18)
		1	12.1% (47)
		2	5.9% (23)
		3	26.8% (104)
		4	50.5% (196)
Combined score	VASOGRADE	Green	15.9% (62)
		Yellow	34.1% (132)
		Red	50.0% (194)
Outcome	Favorable	mRS 0	22.2% (86)
		mRS 1	18.3% (71)
		mRS 2	6.2% (24)
		mRS 3	7.2% (28)
	Unfavorable	mRS 4	7.7% (30)
		mRS 5	4.9% (19)
		mRS 6	33.5% (130)
Treatment	Coiling		57.9% (220)
	Clipping		27.9% (106)
	Other		2.6% (10)
	None		10.9% (25)

The chosen features in feature selection eight demonstrated negligible multicollinearity with VIFs < 3.48 (age 1.08, GCS 1.81, sex 2.77, pupil status 1.44, presence of IVH 2.41, presence of ICH 2.17, presence of MLS 1.85, presence of SDH 1.17, localization of the aneurysm 3.47, BNI score 1.24).

### Predictive performance of existing clinical, radiographic, and combined scores

Predictive performance of established scores for outcome prediction after aSAH ranged between very low (AUC 0.55, original Fisher score) and moderately good (AUC 0.76, Hunt and Hess score and GCS score). The performance of the other scores showed similar ranges. The predictive performances of machine learning models were comparable with traditional GLM methods. For an overview of the performance values and the measures of spread, see Table 2. Detailed results for all additional performance measures are presented in the Supplementary Material (Tables 1–8).

### Predictive performances of the combined set of clinico-radiological features

**Fig. 1:**
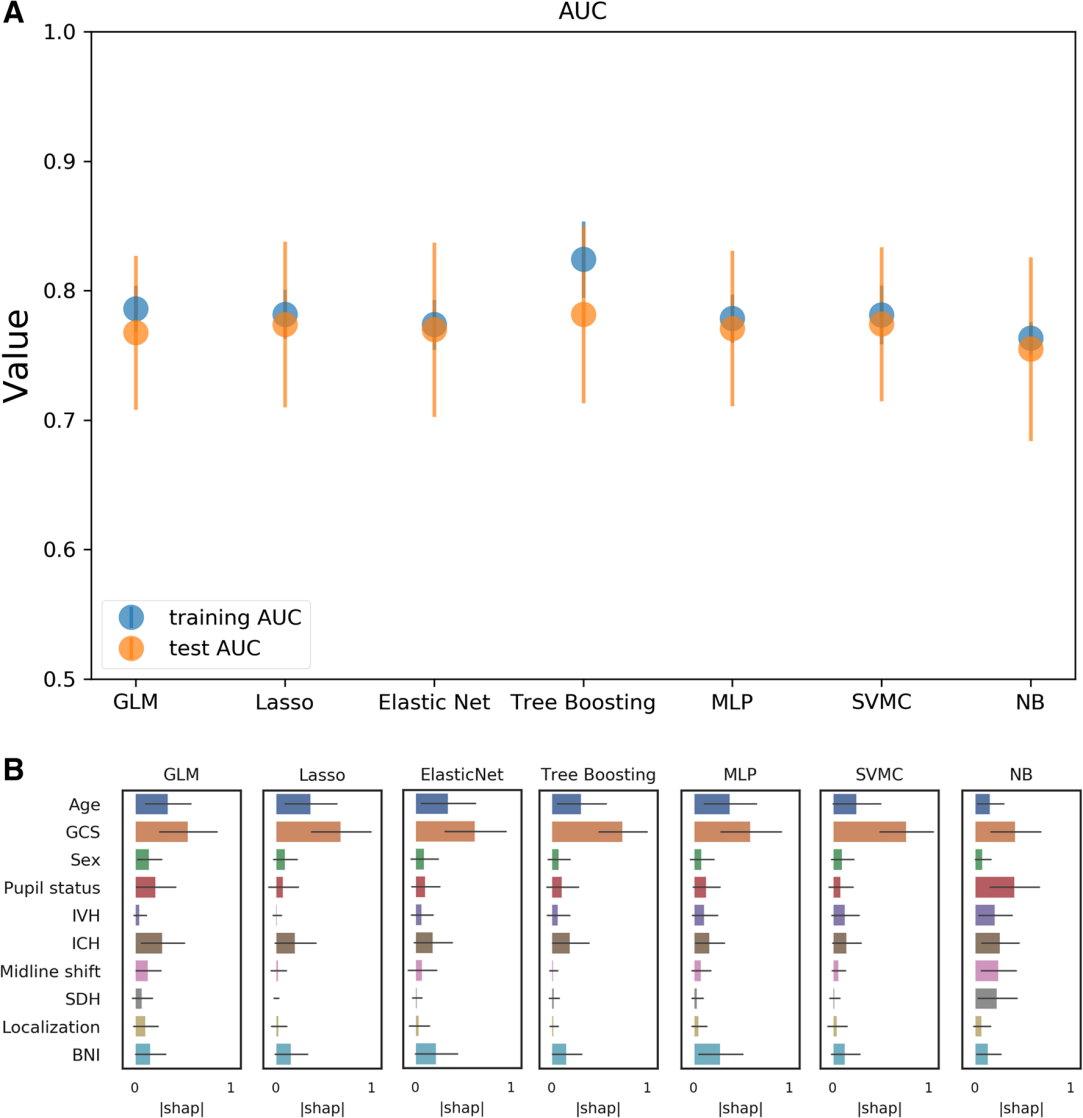
Graphical representation of the performance and feature rating for the clinico-radiological model. **A** The highest test-AUC was 0.78 for the tree boosting model, with the exception of NB (0.75); the other models had a test-AUC value of 0.77. A larger difference between training and test set was observed for the tree boosting model indicative of overfitting. **B** The feature importance rankings consistently identified GCS as the most important factor. Note that model 7, GCS alone, already reached a test-AUC of 0.76. *AUC* area under the curve, *GCS* Glasgow Coma Scale, *GLM* generalized linear model, *IVH* presence of intraventricular hemorrhage, *ICH* presence of intracranial hemorrhage, *NB* Naive Bayes, *MLP* multilayer perceptron, *SDH* presence of subdural hematoma, *SVMC* support vector machine classifier, *BNI* semi-quantitative analysis of the thickness of subarachnoidal blood with respect to the scale introduced by the Barrow Neurological Institute in 2012[7]. The term “localization” refers to the localization of the aneurysm (anterior circulation yes/no)

The combined set of clinical and radiographic features showed an AUC of 0.78 for the tree boosting model and 0.77 for all other models with the exception of the Naive Bayes model (0.75) (Table 3, Fig. 1A). There was no apparent superiority of the combined model over single clinical score models. The feature importance rating identified the GCS score as the most important feature in all models (Fig. 1B). Consistently, the second most important feature was age. The models also assigned importance to BNI and the presence of ICH. The Naive Bayes model was the only model assigning very high importance to pupil status. Results for the additional performance measures are presented in the supplementary material (Suppl. Tables).

**Table 3:** Predictive performance of clinical, radiological, and combined scores as well as the combined feature set (see “Materials and methods” section) for unfavorable patient outcome (mRS 3–6) measured by AUC. Median AUC for the training and the test set (in bold) as well as the interquartile range (IQR) for the test set (in brackets) over 50 shuffles are shown. *AUC* area under the curve, *BNI* Barrow Neurological Institute scale, *GCS* Glasgow Coma Scale, *GLM* generalized linear model, *ICH* intracerebral hemorrhage, *IVH* intraventricular hemorrhage, *MLP* multilayer perceptron, *mRS* Modified Rankin Scale, *NB* Naive Bayes, *WFNS* World Federation of Neurological Societies, *SAH* subarachnoidal hemorrhage, *SDH* subdural hemorrhage, *SVMC* support vector machine classifier

Features	GLM	GLM_Lasso	GLM_elastic_net	CatBoost	MLP	SVMC	NB
Hunt and Hess score	0.75/0.76 (0.07)	0.75/0.75 (0.08)	0.75/0.0.75 (0.08)	0.75/0.76 (0.07)	0.75/0.76 (0.07)	0.75/0.75 (0.07)	0.75/0.76 (0.07)
WFNS score	0.74/0.74 (0.04)	0.74/0.74 (0.04)	0.73/0.74 (0.09)	0.74/0.74 (0.04)	0.74/0.74 (0.04)	0.74/0.74 (0.05)	0.74/0.74 (0.04)
Modified Fisher score	0.65/0.65 (0.07)	0.65/0.65 (0.07)	0.64/0.62 (0.15)	0.65/0.65 (0.07)	0.64/0.65 (0.07)	0.65/0.64 (0.07)	0.65/0.65 (0.07)
Original Fisher score	0.55/0.55 (0.04)	0.55/0.55 (0.05)	0.55/0.52 (0.11)	0.55/0.55 (0.06)	0.55/0.55 (0.04)	0.49/0.47 (0.11)	0.55/0.54 (0.08)
VASOGRADE score	0.72/0.72 (0.07)	0.72/0.72 (0.07)	0.72/0.72 (0.09)	0.72/0.71 (0.06)	0.72/0.72 (0.06)	0.72/0.72 (0.07)	0.72/0.72 (0.07)
BNI score	0.62/0.0.63 (0.06)	0.62/0.63 (0.06)	0.61/0.60 (0.15)	0.62/0.62 (0.07)	0.62/0.62 (0.07)	0.62/0.62 (0.08)	0.62/0.63 (0.06)
GCS score	0.75/0.76 (0.05)	0.75/0.76 (0.05)	0.75/0.75 (0.07)	0.76/0.76 (0.06)	0.75/0.76 (0.06)	0.75/0.76 (0.05)	0.75/0.76 (0.05)
Age, GCS score, sex, pupil status, presence of IVH, presence of ICH, presence of midline shift > 5 mm, presence of SDH, localization anterior circulation or other, BNI score	0.79/0.77 (0.06)	0.78/0.77 (0.06)	0.77/0.77 (0.07)	0.82/0.78 (0.07)	0.78/0.77 (0.06)	0.78/0.77 (0.06)	0.76/0.75 (0.07)

### Estimation of calibration

Based on the Brier score, the calibration was sufficient, ranging from 0.18 to 0.25 over all models. The best calibrated models were the combined set, the GCS model, and the Hunt and Hess score model (Table 4).

**Table 4:** Brier score results for clinical, radiological, and combined scores as well as the combined feature set (see “Materials and methods” section) for prediction of unfavorable patient outcome (mRS 3–6). Median Brier score for the training and the test set (in bold) as well as the interquartile range (IQR) for the test set (in brackets) over 50 shuffles are shown. *AUC* area under the curve, *BNI* Barrow Neurological Institute scale, *GCS* Glasgow Coma Scale, *GLM* generalized linear model, *ICH* intracerebral hemorrhage, *IVH* intraventricular hemorrhage, *MLP* multilayer perceptron, *mRS* Modified Rankin Scale, *NB* Naive Bayes, *WFNS* World Federation of Neurological Surgeons, *SAH* subarachnoidal hemorrhage, *SDH* subdural hemorrhage, *SVMC* support vector machine classifier

Features	GLM	GLM_Lasso	GLM_elastic_net	CatBoost	MLP	SVMC	NB
Hunt and Hess score	0.20/0.20 (0.02)	0.20/0.20 (0.02)	0.24/0.0.24 (0.06)	0.20/0.20 (0.02)	0.21/0.23 (0.05)	0.20/0.20 (0.02)	0.20/0.20 (0.03)
WFNS score	0.20/0.20 (0.02)	0.20/0.20 (0.01)	0.23/0.22 (0.06)	0.20/0.20 (0.02)	0.23/0.24 (0.05)	0.20/0.20 (0.02)	0.20/0.20 (0.02)
Modified Fisher score	0.23/0.23 (0.02)	0.24/0.24 (0.01)	0.25/0.25 (0.03)	0.23/0.23 (0.02)	0.24/0.25 (0.02)	0.23/0.23 (0.01)	0.24/0.24 (0.02)
Original Fisher score	0.25/0.25 (0.01)	0.25/0.25 (0.00)	0.28/0.27 (0.06)	0.25/0.25 (0.01)	0.25/0.25 (0.00)	0.25/0.25 (0.00)	0.25/0.25 (0.02)
VASOGRADE score	0.21/0.21 (0.02)	0.21/0.21 (0.02)	0.24/0.24 (0.07)	0.20/0.20 (0.03)	0.20/0.20 (0.03)	0.20/0.21 (0.03)	0.21/0.21 (0.03)
BNI score	0.24/0.0.24 (0.01)	0.24/0.24 (0.01)	0.25/0.25 (0.04)	0.24/0.24 (0.01)	0.25/0.25 (0.01)	0.24/0.24 (0.01)	0.24/0.24 (0.01)
GCS score	0.20/0.20 (0.02)	0.20/0.20 (0.02)	0.24/0.23 (0.05)	0.19/0.19 (0.03)	0.21/0.23 (0.05)	0.20/0.20 (0.02)	0.20/0.20 (0.03)
Age, GCS score, sex, pupil status, presence of IVH, presence of ICH, presence of midline shift > 5 mm, presence of SDH, localization anterior circulation or other, BNI score	0.19/0.19 (0.03)	0.19/0.19 (0.03)	0.20/0.21 (0.03)	0.18/0.19 (0.03)	0.19/0.20 (0.04)	0.19/0.19 (0.03)	0.24/0.23 (0.07)

## Discussion

In this study on aSAH outcome prediction, we observed moderately good performances of ML methods using traditional clinico-radiographic features available on admission. There was no difference in performance between any of the applied techniques, especially not between the traditional techniques (GLM), and the most modern techniques (CatBoost tree boosting, MLP). Furthermore, we observed no superiority of the examined ML techniques over the best performing clinical scores on admission (GCS and Hunt and Hess). Thus, we could not establish a relevant advantage of state-of-the-art ML methods for aSAH outcome prediction by using patient-specific clinical and radiographic features available on admission.

Outcome prediction in aSAH is usually conducted using traditional clinical and radiological scores on patient admission. Outcome prediction models find use in counseling of patients and their relatives as well as in the selection of treatment strategies. Especially in the presence of an ongoing global pandemic, precise predictions of outcomes in critically ill patients may help allocate scarce medical resources [4, 11, 33]. Therefore, the transparency, comparability, and reproducibility of outcome prediction models are of utmost importance. Recently, the comparability of clinical, radiographic, and combined scores in the same patient cohort was established. A combination of clinical and radiographic elements within single combined scores (VASOGRADE) did not show a significant improvement of predictive score performance regarding the prediction of angiographic vasospasm, cerebral infarction, and unfavorable outcome [9]. Another study showed that even patients with the highest Hunt and Hess score (V) have favorable outcome in 26 % of cases in a retrospective multicenter series [36].

The majority of previously established aSAH outcome prediction models are based on neurological deficits on admission and radiographic features, such as thickness of subarachnoid blood clots and the presence of IVH or ICH. However, more recent evidence suggests that other factors play a role in precise outcome prediction, such as patient age, pupil status, and aneurysm size and location[21, 29]. The inclusion of a high number of variables is one of the main strengths of ML approaches. In numerous medical fields, ML-based prediction models were shown to be superior to traditional techniques [12, 38]. In neurosurgery, ML prediction models have been evaluated for a variety of pathologies with variable predictive performances (AUC 0.71 to 0.96) [26]. In the prediction of the occurrence of shunt-dependent hydrocephalus after aSAH, ML methods proved to be superior to traditional methods [24]. They included dynamic variables such as infections, treatment timing from symptom onset, and fever onset. In predicting early complications after intracranial tumor surgery, ML methods showed slight superiority over conventional traditional methods [30]. In our present study, we applied—amongst others—two of the most promising state-of-the-art ML techniques to predict functional outcome after aSAH: tree boosting and ANN. Both have shown considerable advances over traditional linear or logistic regression techniques in the past [12, 38], even though traceability and comparability across different studies is reduced by substantial heterogeneity of clinical questions, input and output variables, and applied algorithms [19, 24, 26, 30].

To maintain transparency and comparability to existing models, our current approach uses established scoring systems. We applied a variety of ML techniques to the same dataset and acquired rather equivalent results regarding predictive power, sensitivity, and specificity but some difference regarding feature rating. Our analyses showed no superiority of any of the examined ML methods over traditional methods for aSAH outcome prediction. A combined set of relevant radiological and clinical features showed only a small superiority to simple and established clinical scores (e.g., tree boosting on the combined features vs. Hunt and Hess and GCS alone). This was also shown for a decision tree model that reached similar accuracy than logistic regression in another study [7]. Notably, one of the main advantages of ML methods is their ability to capture even weak interactions between variables to make predictions. Nevertheless, our findings suggest that currently available scores and variables used to feed ML-based prediction models for aSAH may not contain enough information to improve the accuracy of outcome predictions.

Thus, it is warranted to explore the addition of other features available on patient admission in future works on early prediction models. These features could include laboratory data, imaging source data, and comorbidities. Also, events occurring during later phases of the course of aSAH, such as infectious diseases (e.g., pneumonia, meningitis), or cardiovascular complications (e.g., Takotsubo myocarditis) may be added over time to improve predictive performances [5, 16, 20]. General scores with special focus on physiology parameters shown to predict the course of intensive care treatment like the APACHE or SOFA scores could be added as well. To our knowledge, only one other work used ML techniques to predict outcome after aSAH [19]. While the analysis was performed in a large prospective multicenter cohort of aSAH patients, in that work outdated methodology, selection of features beyond admission, the lack of reported AUC, and Glasgow Outcome Score as the outcome measure make the models clinically less applicable and not comparable to our work.

## Limitations of our study

Limitations of our study include the retrospective, single-center study design impacting the availability of features. Our patient sample is medium sized compared to existing studies applying ML methods for aSAH outcome prediction [7, 19]. However, a selection bias applies for most other studies that analyze aSAH as they are often taken from multicenter trial data with specific study protocols and inclusion/exclusion criteria. Our data represent real-world data from a single high-volume center in Germany. Our results may therefore not be generalized to other centers or countries [10]. Mean/mode imputation is not a state-of-the-art imputation method. State-of-the-art imputation methods are currently not tailored to predictive modelling, i.e., the transfer of imputation models from training to test set is not straightforward. However, given the very low ratio of missing values in our study, we deem this issue negligible and encourage the development of methods allowing the transfer of imputation models tailored to predictive modelling in Python. The very small imbalance in dichotomized outcome numbers may cause negligible bias. It is thus acknowledged but did not warrant a sub-sampling approach limiting the available data for model training.

## Conclusion

Our study applies ML techniques for functional outcome prediction after aSAH on the basis of clinico-radiographic variables available at patient admission. We could demonstrate that the predictive performance of ML techniques was comparable but not superior to established traditional methods and established clinical scores. In conclusion, our findings make a compelling case for the exploration of new input variables other than traditional clinico-radiographic features to achieve a higher accuracy for outcome prediction in aSAH in the future.

## Supplementary Information

ESM 1 (DOCX 64 kb)

## Abbreviations

ANN: Artificial neural net
aSAH: Aneurysmal subarachnoid hemorrhage
AUC: Area under the curve
aVS: Angiographic vasospasm,
BNI: Barrow Neurological Institute scale
CI: Cerebral infarction
CT: Computed tomography
DIND: Delayed ischemic neurological deficit
DL: Deep learning
DSA: Digital subtraction angiogram
GCS: Glasgow Coma Scale
GLM: Generalized linear model
ICH: Intracerebral hemorrhage
ICU: Intensive care unit
IVH: Intraventricular hemorrhage
ML: Machine learning
MLP: Multilayer perceptron artificial neural net
mRS: Modified Rankin Scale
NB: Naive Bayes
ROC: Receiver operating characteristics
SVMC: Support vector machine classifier
WFNS: World Federation of Neurological Societies
