# Supplementary material for: Outcome prediction in aneurysmal subarachnoid hemorrhage: a comparison of machine learning methods and established clinico-radiological scores
Source: Neurosurg Rev. 2021 Jan 20;44(5):2837–46. doi: 10.1007/s10143-020-01453-6 (PMC8490233; doi:10.1007/s10143-020-01453-6)
Supplement: Supplementary file 1 — (DOCX 64 kb) [file 10143_2020_1453_MOESM1_ESM.docx]

Feature selection 1 Hunt and Hess Score

| **Model** | **Value** | **AUC (training)** | **AUC (test)** | **accuracy (training)** | **accuracy (test)** | **average class accuracy (training)** | **average class accuracy (test)** | **f1 (training)** | **f1 (test)** | **precision PPV (training)** | **precision PPV (test)** | **NPV (training)** | **NPV (test)** | **recall sensitivity (training)** | **recall sensitivity (test)** | **specificity (training)** | **specificity (test)** | **brier score loss (training)** | **brier score loss (test)** |
| --- | --- | --- | --- | --- | --- | --- | --- | --- | --- | --- | --- | --- | --- | --- | --- | --- | --- | --- | --- |
| GLM | median | 0.75 | 0.76 | 0.70 | 0.71 | 0.70 | 0.70 | 0.71 | 0.71 | 0.65 | 0.65 | 0.77 | 0.77 | 0.78 | 0.79 | 0.62 | 0.64 | 0.20 | 0.20 |
| GLM | iqr | 0.02 | 0.07 | 0.01 | 0.05 | 0.01 | 0.06 | 0.01 | 0.05 | 0.01 | 0.05 | 0.02 | 0.07 | 0.02 | 0.08 | 0.02 | 0.07 | 0.01 | 0.02 |
| Lasso | median | 0.75 | 0.75 | 0.70 | 0.71 | 0.70 | 0.69 | 0.71 | 0.71 | 0.65 | 0.64 | 0.77 | 0.77 | 0.78 | 0.78 | 0.62 | 0.64 | 0.20 | 0.20 |
| Lasso | iqr | 0.02 | 0.08 | 0.01 | 0.06 | 0.01 | 0.07 | 0.01 | 0.05 | 0.01 | 0.05 | 0.02 | 0.07 | 0.02 | 0.08 | 0.02 | 0.07 | 0.01 | 0.02 |
| ElasticNet | median | 0.75 | 0.75 | 0.70 | 0.69 | 0.70 | 0.69 | 0.71 | 0.70 | 0.65 | 0.64 | 0.77 | 0.77 | 0.78 | 0.78 | 0.62 | 0.64 | 0.24 | 0.24 |
| ElasticNet | iqr | 0.02 | 0.08 | 0.02 | 0.06 | 0.02 | 0.06 | 0.01 | 0.06 | 0.02 | 0.06 | 0.02 | 0.07 | 0.02 | 0.10 | 0.02 | 0.10 | 0.03 | 0.06 |
| Tree boosting | median | 0.75 | 0.76 | 0.71 | 0.71 | 0.70 | 0.69 | 0.68 | 0.68 | 0.70 | 0.69 | 0.71 | 0.72 | 0.64 | 0.67 | 0.77 | 0.76 | 0.20 | 0.20 |
| Tree boosting | iqr | 0.02 | 0.07 | 0.02 | 0.06 | 0.02 | 0.07 | 0.03 | 0.05 | 0.03 | 0.09 | 0.03 | 0.05 | 0.05 | 0.10 | 0.03 | 0.10 | 0.00 | 0.02 |
| MLP | median | 0.75 | 0.76 | 0.70 | 0.69 | 0.69 | 0.67 | 0.66 | 0.65 | 0.70 | 0.67 | 0.70 | 0.70 | 0.62 | 0.60 | 0.78 | 0.82 | 0.21 | 0.23 |
| MLP | iqr | 0.02 | 0.07 | 0.17 | 0.19 | 0.70 | 0.72 | 0.67 | 0.70 | 0.71 | 0.73 | 0.18 | 0.19 | 0.63 | 0.67 | 0.24 | 0.24 | 0.05 | 0.05 |
| SVMC | median | 0.75 | 0.75 | 0.70 | 0.71 | 0.70 | 0.69 | 0.68 | 0.69 | 0.70 | 0.68 | 0.72 | 0.73 | 0.65 | 0.67 | 0.76 | 0.74 | 0.20 | 0.20 |
| SVMC | iqr | 0.02 | 0.07 | 0.01 | 0.06 | 0.02 | 0.07 | 0.04 | 0.07 | 0.06 | 0.10 | 0.06 | 0.07 | 0.14 | 0.15 | 0.14 | 0.14 | 0.01 | 0.02 |
| NB | median | 0.75 | 0.76 | 0.70 | 0.72 | 0.69 | 0.70 | 0.67 | 0.68 | 0.71 | 0.71 | 0.70 | 0.72 | 0.63 | 0.64 | 0.77 | 0.79 | 0.20 | 0.20 |
| NB | iqr | 0.02 | 0.07 | 0.02 | 0.06 | 0.01 | 0.06 | 0.01 | 0.06 | 0.02 | 0.08 | 0.01 | 0.05 | 0.02 | 0.08 | 0.02 | 0.07 | 0.01 | 0.03 |

Feature selection 2 WFNS score

| **Model** | **Value** | **AUC (training)** | **AUC (test)** | **accuracy (training)** | **accuracy (test)** | **average class accuracy (training)** | **average class accuracy (test)** | **f1 (training)** | **f1 (test)** | **precision PPV (training)** | **precision PPV (test)** | **NPV (training)** | **NPV (test)** | **recall sensitivity (training)** | **recall sensitivity (test)** | **specificity (training)** | **specificity (test)** | **brier score loss (training)** | **brier score loss (test)** |
| --- | --- | --- | --- | --- | --- | --- | --- | --- | --- | --- | --- | --- | --- | --- | --- | --- | --- | --- | --- |
| GLM | median | 0.74 | 0.74 | 0.71 | 0.72 | 0.71 | 0.72 | 0.70 | 0.71 | 0.68 | 0.69 | 0.74 | 0.76 | 0.72 | 0.72 | 0.70 | 0.71 | 0.20 | 0.20 |
| GLM | iqr | 0.01 | 0.04 | 0.01 | 0.05 | 0.01 | 0.05 | 0.01 | 0.06 | 0.02 | 0.05 | 0.01 | 0.06 | 0.01 | 0.10 | 0.02 | 0.09 | 0.00 | 0.02 |
| Lasso | median | 0.74 | 0.74 | 0.71 | 0.72 | 0.71 | 0.72 | 0.70 | 0.71 | 0.68 | 0.69 | 0.74 | 0.76 | 0.72 | 0.72 | 0.70 | 0.71 | 0.20 | 0.20 |
| Lasso | iqr | 0.01 | 0.04 | 0.01 | 0.05 | 0.01 | 0.05 | 0.01 | 0.06 | 0.02 | 0.05 | 0.01 | 0.06 | 0.01 | 0.10 | 0.02 | 0.09 | 0.00 | 0.01 |
| ElasticNet | median | 0.73 | 0.74 | 0.71 | 0.71 | 0.71 | 0.69 | 0.70 | 0.68 | 0.68 | 0.68 | 0.74 | 0.73 | 0.72 | 0.72 | 0.70 | 0.69 | 0.23 | 0.22 |
| ElasticNet | iqr | 0.02 | 0.09 | 0.02 | 0.06 | 0.02 | 0.07 | 0.03 | 0.08 | 0.03 | 0.09 | 0.03 | 0.08 | 0.03 | 0.14 | 0.03 | 0.12 | 0.04 | 0.06 |
| Tree boosting | median | 0.74 | 0.74 | 0.72 | 0.73 | 0.71 | 0.73 | 0.70 | 0.71 | 0.70 | 0.70 | 0.73 | 0.75 | 0.69 | 0.72 | 0.74 | 0.74 | 0.20 | 0.20 |
| Tree boosting | iqr | 0.01 | 0.04 | 0.01 | 0.04 | 0.01 | 0.05 | 0.02 | 0.06 | 0.01 | 0.06 | 0.01 | 0.05 | 0.02 | 0.08 | 0.02 | 0.07 | 0.00 | 0.02 |
| MLP | median | 0.74 | 0.74 | 0.71 | 0.69 | 0.70 | 0.68 | 0.69 | 0.65 | 0.70 | 0.66 | 0.72 | 0.70 | 0.68 | 0.61 | 0.76 | 0.79 | 0.23 | 0.24 |
| MLP | iqr | 0.01 | 0.04 | 0.19 | 0.20 | 0.72 | 0.73 | 0.70 | 0.71 | 0.70 | 0.71 | 0.20 | 0.21 | 0.70 | 0.72 | 0.26 | 0.28 | 0.05 | 0.05 |
| SVMC | median | 0.74 | 0.74 | 0.71 | 0.73 | 0.71 | 0.72 | 0.70 | 0.71 | 0.69 | 0.70 | 0.74 | 0.75 | 0.71 | 0.72 | 0.72 | 0.74 | 0.20 | 0.20 |
| SVMC | iqr | 0.02 | 0.05 | 0.01 | 0.05 | 0.01 | 0.05 | 0.02 | 0.06 | 0.02 | 0.06 | 0.02 | 0.05 | 0.03 | 0.08 | 0.04 | 0.07 | 0.00 | 0.02 |
| NB | median | 0.74 | 0.74 | 0.72 | 0.73 | 0.71 | 0.73 | 0.70 | 0.71 | 0.70 | 0.70 | 0.73 | 0.75 | 0.69 | 0.72 | 0.74 | 0.74 | 0.20 | 0.20 |
| NB | iqr | 0.01 | 0.04 | 0.01 | 0.04 | 0.01 | 0.05 | 0.02 | 0.06 | 0.01 | 0.06 | 0.01 | 0.05 | 0.02 | 0.08 | 0.02 | 0.07 | 0.00 | 0.02 |

Feature Selection 3 Modified Fisher Score

| **Model** | **Value** | **AUC (training)** | **AUC (test)** | **accuracy (training)** | **accuracy (test)** | **average class accuracy (training)** | **average class accuracy (test)** | **f1 (training)** | **f1 (test)** | **precision PPV (training)** | **precision PPV (test)** | **NPV (training)** | **NPV (test)** | **recall sensitivity (training)** | **recall sensitivity (test)** | **specificity (training)** | **specificity (test)** | **brier score loss (training)** | **brier score loss (test)** |
| --- | --- | --- | --- | --- | --- | --- | --- | --- | --- | --- | --- | --- | --- | --- | --- | --- | --- | --- | --- |
| GLM | median | 0.65 | 0.65 | 0.64 | 0.64 | 0.64 | 0.63 | 0.62 | 0.61 | 0.61 | 0.60 | 0.67 | 0.67 | 0.64 | 0.64 | 0.64 | 0.62 | 0.23 | 0.23 |
| GLM | iqr | 0.02 | 0.07 | 0.02 | 0.07 | 0.02 | 0.07 | 0.02 | 0.06 | 0.02 | 0.06 | 0.01 | 0.06 | 0.02 | 0.08 | 0.02 | 0.07 | 0.00 | 0.02 |
| Lasso | median | 0.65 | 0.65 | 0.64 | 0.64 | 0.64 | 0.63 | 0.62 | 0.61 | 0.61 | 0.60 | 0.67 | 0.67 | 0.64 | 0.64 | 0.64 | 0.62 | 0.24 | 0.24 |
| Lasso | iqr | 0.02 | 0.07 | 0.02 | 0.07 | 0.02 | 0.07 | 0.02 | 0.06 | 0.02 | 0.06 | 0.01 | 0.06 | 0.02 | 0.08 | 0.02 | 0.07 | 0.00 | 0.01 |
| ElasticNet | median | 0.64 | 0.62 | 0.63 | 0.62 | 0.63 | 0.62 | 0.61 | 0.60 | 0.60 | 0.58 | 0.66 | 0.66 | 0.63 | 0.62 | 0.63 | 0.62 | 0.25 | 0.25 |
| ElasticNet | iqr | 0.12 | 0.15 | 0.09 | 0.12 | 0.20 | 0.21 | 0.20 | 0.20 | 0.20 | 0.20 | 0.11 | 0.15 | 0.19 | 0.17 | 0.02 | 0.10 | 0.04 | 0.03 |
| Tree boosting | median | 0.65 | 0.65 | 0.64 | 0.64 | 0.64 | 0.63 | 0.62 | 0.61 | 0.61 | 0.60 | 0.67 | 0.67 | 0.64 | 0.64 | 0.64 | 0.62 | 0.23 | 0.23 |
| Tree boosting | iqr | 0.01 | 0.07 | 0.02 | 0.07 | 0.02 | 0.07 | 0.02 | 0.06 | 0.02 | 0.06 | 0.01 | 0.06 | 0.02 | 0.08 | 0.02 | 0.07 | 0.00 | 0.02 |
| MLP | median | 0.64 | 0.65 | 0.59 | 0.60 | 0.46 | 0.53 | 0.42 | 0.52 | 0.60 | 0.60 | 0.58 | 0.61 | 0.32 | 0.43 | 0.83 | 0.81 | 0.24 | 0.25 |
| MLP | iqr | 0.02 | 0.07 | 0.08 | 0.11 | 0.49 | 0.51 | 0.48 | 0.49 | 0.04 | 0.14 | 0.12 | 0.11 | 0.56 | 0.57 | 0.34 | 0.33 | 0.02 | 0.02 |
| SVMC | median | 0.65 | 0.64 | 0.59 | 0.60 | 0.40 | 0.44 | 0.37 | 0.40 | 0.64 | 0.63 | 0.57 | 0.59 | 0.26 | 0.29 | 0.88 | 0.86 | 0.23 | 0.23 |
| SVMC | iqr | 0.02 | 0.07 | 0.04 | 0.05 | 0.25 | 0.13 | 0.25 | 0.19 | 0.04 | 0.10 | 0.07 | 0.05 | 0.34 | 0.26 | 0.19 | 0.20 | 0.00 | 0.01 |
| NB | median | 0.65 | 0.65 | 0.59 | 0.60 | 0.44 | 0.47 | 0.40 | 0.43 | 0.62 | 0.63 | 0.58 | 0.59 | 0.30 | 0.33 | 0.84 | 0.83 | 0.24 | 0.24 |
| NB | iqr | 0.02 | 0.07 | 0.01 | 0.05 | 0.02 | 0.10 | 0.03 | 0.10 | 0.03 | 0.10 | 0.01 | 0.04 | 0.02 | 0.08 | 0.02 | 0.07 | 0.00 | 0.02 |

Feature selection 4 Original Fisher score

| **Model** | **Value** | **AUC (training)** | **AUC (test)** | **accuracy (training)** | **accuracy (test)** | **average class accuracy (training)** | **average class accuracy (test)** | **f1 (training)** | **f1 (test)** | **precision PPV (training)** | **precision PPV (test)** | **NPV (training)** | **NPV (test)** | **recall sensitivity (training)** | **recall sensitivity (test)** | **specificity (training)** | **specificity (test)** | **brier score loss (training)** | **brier score loss (test)** |
| --- | --- | --- | --- | --- | --- | --- | --- | --- | --- | --- | --- | --- | --- | --- | --- | --- | --- | --- | --- |
| GLM | median | 0.55 | 0.55 | 0.56 | 0.56 | 0.26 | 0.24 | 0.24 | 0.23 | 0.63 | 0.62 | 0.55 | 0.56 | 0.15 | 0.14 | 0.92 | 0.93 | 0.25 | 0.25 |
| GLM | iqr | 0.01 | 0.04 | 0.01 | 0.05 | 0.02 | 0.09 | 0.02 | 0.09 | 0.05 | 0.21 | 0.01 | 0.02 | 0.01 | 0.06 | 0.01 | 0.05 | 0.00 | 0.01 |
| Lasso | median | 0.55 | 0.55 | 0.56 | 0.56 | 0.26 | 0.24 | 0.24 | 0.23 | 0.63 | 0.62 | 0.55 | 0.56 | 0.15 | 0.14 | 0.92 | 0.93 | 0.25 | 0.25 |
| Lasso | iqr | 0.01 | 0.05 | 0.01 | 0.05 | 0.03 | 0.09 | 0.02 | 0.09 | 0.05 | 0.21 | 0.01 | 0.03 | 0.02 | 0.06 | 0.01 | 0.05 | 0.00 | 0.00 |
| ElasticNet | median | 0.55 | 0.52 | 0.55 | 0.54 | 0.25 | 0.20 | 0.25 | 0.24 | 0.61 | 0.50 | 0.55 | 0.55 | 0.16 | 0.15 | 0.92 | 0.90 | 0.28 | 0.27 |
| ElasticNet | iqr | 0.10 | 0.11 | 0.11 | 0.09 | 0.11 | 0.09 | 0.34 | 0.35 | 0.19 | 0.24 | 0.14 | 0.06 | 0.68 | 0.65 | 0.84 | 0.83 | 0.05 | 0.06 |
| Tree boosting | median | 0.55 | 0.55 | 0.56 | 0.56 | 0.26 | 0.24 | 0.24 | 0.23 | 0.63 | 0.62 | 0.55 | 0.56 | 0.15 | 0.14 | 0.92 | 0.93 | 0.25 | 0.25 |
| Tree boosting | iqr | 0.01 | 0.06 | 0.01 | 0.05 | 0.02 | 0.09 | 0.02 | 0.09 | 0.05 | 0.21 | 0.01 | 0.02 | 0.01 | 0.06 | 0.01 | 0.05 | 0.00 | 0.01 |
| MLP | median | 0.55 | 0.55 | 0.53 | 0.54 | 0.12 | 0.05 | 0.11 | 0.05 | 0.51 | 0.33 | 0.54 | 0.54 | 0.06 | 0.03 | 0.97 | 0.98 | 0.25 | 0.25 |
| MLP | iqr | 0.01 | 0.04 | 0.02 | 0.01 | 0.16 | 0.20 | 0.16 | 0.18 | 0.62 | 0.54 | 0.01 | 0.01 | 0.09 | 0.11 | 0.05 | 0.07 | 0.00 | 0.00 |
| SVMC | median | 0.49 | 0.47 | 0.53 | 0.54 | 0.00 | 0.00 | 0.00 | 0.00 | 0.00 | 0.00 | 0.53 | 0.54 | 0.00 | 0.00 | 1.00 | 1.00 | 0.25 | 0.25 |
| SVMC | iqr | 0.10 | 0.11 | 0.01 | 0.01 | 0.15 | 0.20 | 0.15 | 0.19 | 0.59 | 0.54 | 0.01 | 0.01 | 0.08 | 0.11 | 0.05 | 0.07 | 0.00 | 0.00 |
| NB | median | 0.55 | 0.54 | 0.56 | 0.56 | 0.26 | 0.24 | 0.24 | 0.23 | 0.63 | 0.62 | 0.55 | 0.56 | 0.15 | 0.14 | 0.92 | 0.93 | 0.25 | 0.25 |
| NB | iqr | 0.02 | 0.08 | 0.01 | 0.05 | 0.02 | 0.08 | 0.02 | 0.08 | 0.06 | 0.20 | 0.01 | 0.02 | 0.01 | 0.06 | 0.01 | 0.05 | 0.00 | 0.02 |

Feature selection 5 Vasograde score

| **Model** | **Value** | **AUC (training)** | **AUC (test)** | **accuracy (training)** | **accuracy (test)** | **average class accuracy (training)** | **average class accuracy (test)** | **f1 (training)** | **f1 (test)** | **precision PPV (training)** | **precision PPV (test)** | **NPV (training)** | **NPV (test)** | **recall sensitivity (training)** | **recall sensitivity (test)** | **specificity (training)** | **specificity (test)** | **brier score loss (training)** | **brier score loss (test)** |
| --- | --- | --- | --- | --- | --- | --- | --- | --- | --- | --- | --- | --- | --- | --- | --- | --- | --- | --- | --- |
| GLM | median | 0.72 | 0.72 | 0.72 | 0.72 | 0.72 | 0.72 | 0.71 | 0.71 | 0.68 | 0.68 | 0.75 | 0.76 | 0.73 | 0.72 | 0.70 | 0.71 | 0.21 | 0.21 |
| GLM | iqr | 0.02 | 0.07 | 0.02 | 0.07 | 0.02 | 0.07 | 0.02 | 0.07 | 0.02 | 0.06 | 0.02 | 0.07 | 0.03 | 0.08 | 0.02 | 0.05 | 0.01 | 0.02 |
| Lasso | median | 0.72 | 0.72 | 0.72 | 0.72 | 0.72 | 0.72 | 0.71 | 0.71 | 0.68 | 0.68 | 0.75 | 0.76 | 0.73 | 0.72 | 0.70 | 0.71 | 0.21 | 0.21 |
| Lasso | iqr | 0.02 | 0.07 | 0.02 | 0.07 | 0.02 | 0.07 | 0.02 | 0.07 | 0.02 | 0.06 | 0.02 | 0.07 | 0.03 | 0.08 | 0.02 | 0.05 | 0.01 | 0.02 |
| ElasticNet | median | 0.72 | 0.72 | 0.72 | 0.71 | 0.72 | 0.71 | 0.71 | 0.70 | 0.68 | 0.68 | 0.75 | 0.75 | 0.72 | 0.72 | 0.70 | 0.69 | 0.24 | 0.24 |
| ElasticNet | iqr | 0.02 | 0.09 | 0.02 | 0.09 | 0.02 | 0.10 | 0.02 | 0.11 | 0.02 | 0.07 | 0.02 | 0.10 | 0.03 | 0.13 | 0.02 | 0.07 | 0.04 | 0.07 |
| Tree boosting | median | 0.72 | 0.71 | 0.72 | 0.72 | 0.72 | 0.72 | 0.71 | 0.70 | 0.69 | 0.69 | 0.75 | 0.76 | 0.73 | 0.72 | 0.71 | 0.71 | 0.20 | 0.20 |
| Tree boosting | iqr | 0.01 | 0.06 | 0.02 | 0.07 | 0.02 | 0.07 | 0.02 | 0.07 | 0.01 | 0.06 | 0.02 | 0.07 | 0.03 | 0.10 | 0.01 | 0.05 | 0.00 | 0.03 |
| MLP | median | 0.72 | 0.72 | 0.71 | 0.72 | 0.72 | 0.72 | 0.71 | 0.70 | 0.68 | 0.68 | 0.75 | 0.75 | 0.73 | 0.72 | 0.70 | 0.71 | 0.20 | 0.20 |
| MLP | iqr | 0.02 | 0.06 | 0.02 | 0.06 | 0.02 | 0.08 | 0.02 | 0.09 | 0.02 | 0.07 | 0.02 | 0.09 | 0.03 | 0.11 | 0.02 | 0.05 | 0.01 | 0.03 |
| SVMC | median | 0.72 | 0.72 | 0.71 | 0.70 | 0.71 | 0.70 | 0.70 | 0.69 | 0.69 | 0.68 | 0.75 | 0.74 | 0.72 | 0.69 | 0.72 | 0.71 | 0.20 | 0.21 |
| SVMC | iqr | 0.02 | 0.07 | 0.12 | 0.11 | 0.32 | 0.30 | 0.33 | 0.31 | 0.02 | 0.06 | 0.17 | 0.17 | 0.48 | 0.45 | 0.20 | 0.18 | 0.01 | 0.03 |
| NB | median | 0.72 | 0.72 | 0.72 | 0.72 | 0.72 | 0.72 | 0.71 | 0.70 | 0.69 | 0.69 | 0.75 | 0.76 | 0.73 | 0.72 | 0.71 | 0.71 | 0.21 | 0.21 |
| NB | iqr | 0.02 | 0.07 | 0.02 | 0.07 | 0.02 | 0.07 | 0.02 | 0.07 | 0.01 | 0.06 | 0.02 | 0.07 | 0.03 | 0.10 | 0.01 | 0.05 | 0.01 | 0.03 |

Feature selection 6 BNI Score

| **Model** | **Value** | **AUC (training)** | **AUC (test)** | **accuracy (training)** | **accuracy (test)** | **average class accuracy (training)** | **average class accuracy (test)** | **f1 (training)** | **f1 (test)** | **precision PPV (training)** | **precision PPV (test)** | **NPV (training)** | **NPV (test)** | **recall sensitivity (training)** | **recall sensitivity (test)** | **specificity (training)** | **specificity (test)** | **brier score loss (training)** | **brier score loss (test)** |
| --- | --- | --- | --- | --- | --- | --- | --- | --- | --- | --- | --- | --- | --- | --- | --- | --- | --- | --- | --- |
| GLM | median | 0.62 | 0.63 | 0.58 | 0.58 | 0.58 | 0.57 | 0.58 | 0.58 | 0.55 | 0.54 | 0.62 | 0.62 | 0.61 | 0.61 | 0.56 | 0.55 | 0.24 | 0.24 |
| GLM | iqr | 0.02 | 0.06 | 0.02 | 0.06 | 0.02 | 0.07 | 0.02 | 0.06 | 0.02 | 0.05 | 0.02 | 0.06 | 0.03 | 0.10 | 0.02 | 0.10 | 0.00 | 0.01 |
| Lasso | median | 0.62 | 0.63 | 0.58 | 0.58 | 0.58 | 0.57 | 0.58 | 0.58 | 0.55 | 0.54 | 0.62 | 0.62 | 0.61 | 0.61 | 0.56 | 0.55 | 0.24 | 0.24 |
| Lasso | iqr | 0.02 | 0.06 | 0.02 | 0.06 | 0.02 | 0.07 | 0.02 | 0.06 | 0.02 | 0.05 | 0.02 | 0.06 | 0.03 | 0.10 | 0.02 | 0.10 | 0.00 | 0.01 |
| ElasticNet | median | 0.61 | 0.60 | 0.58 | 0.56 | 0.58 | 0.55 | 0.57 | 0.55 | 0.54 | 0.52 | 0.62 | 0.61 | 0.61 | 0.58 | 0.56 | 0.55 | 0.25 | 0.25 |
| ElasticNet | iqr | 0.12 | 0.15 | 0.05 | 0.08 | 0.17 | 0.16 | 0.18 | 0.16 | 0.16 | 0.15 | 0.09 | 0.10 | 0.21 | 0.22 | 0.05 | 0.12 | 0.04 | 0.04 |
| Tree boosting | median | 0.62 | 0.62 | 0.59 | 0.56 | 0.58 | 0.53 | 0.57 | 0.52 | 0.56 | 0.54 | 0.62 | 0.59 | 0.59 | 0.54 | 0.58 | 0.57 | 0.24 | 0.24 |
| Tree boosting | iqr | 0.02 | 0.07 | 0.01 | 0.05 | 0.14 | 0.16 | 0.17 | 0.21 | 0.06 | 0.06 | 0.05 | 0.06 | 0.31 | 0.35 | 0.27 | 0.33 | 0.00 | 0.01 |
| MLP | median | 0.62 | 0.62 | 0.57 | 0.54 | 0.41 | 0.28 | 0.37 | 0.25 | 0.55 | 0.49 | 0.57 | 0.55 | 0.27 | 0.17 | 0.84 | 0.89 | 0.25 | 0.25 |
| MLP | iqr | 0.02 | 0.07 | 0.05 | 0.06 | 0.54 | 0.52 | 0.53 | 0.49 | 0.60 | 0.61 | 0.06 | 0.07 | 0.52 | 0.47 | 0.36 | 0.30 | 0.01 | 0.01 |
| SVMC | median | 0.62 | 0.62 | 0.55 | 0.56 | 0.18 | 0.26 | 0.17 | 0.24 | 0.61 | 0.60 | 0.54 | 0.55 | 0.10 | 0.15 | 0.95 | 0.95 | 0.24 | 0.24 |
| SVMC | iqr | 0.02 | 0.08 | 0.04 | 0.04 | 0.30 | 0.29 | 0.27 | 0.25 | 0.06 | 0.27 | 0.04 | 0.03 | 0.22 | 0.22 | 0.12 | 0.16 | 0.01 | 0.01 |
| NB | median | 0.62 | 0.63 | 0.59 | 0.56 | 0.58 | 0.53 | 0.57 | 0.52 | 0.56 | 0.54 | 0.61 | 0.59 | 0.60 | 0.54 | 0.58 | 0.60 | 0.24 | 0.24 |
| NB | iqr | 0.02 | 0.06 | 0.02 | 0.06 | 0.14 | 0.16 | 0.18 | 0.19 | 0.05 | 0.09 | 0.05 | 0.07 | 0.31 | 0.35 | 0.27 | 0.33 | 0.00 | 0.01 |

Feature selection 7 GCS Score

| **Model** | **Value** | **AUC (training)** | **AUC (test)** | **accuracy (training)** | **accuracy (test)** | **average class accuracy (training)** | **average class accuracy (test)** | **f1 (training)** | **f1 (test)** | **precision PPV (training)** | **precision PPV (test)** | **NPV (training)** | **NPV (test)** | **recall sensitivity (training)** | **recall sensitivity (test)** | **specificity (training)** | **specificity (test)** | **brier score loss (training)** | **brier score loss (test)** |
| --- | --- | --- | --- | --- | --- | --- | --- | --- | --- | --- | --- | --- | --- | --- | --- | --- | --- | --- | --- |
| GLM | median | 0.75 | 0.76 | 0.69 | 0.70 | 0.69 | 0.69 | 0.70 | 0.71 | 0.64 | 0.64 | 0.77 | 0.78 | 0.79 | 0.81 | 0.61 | 0.62 | 0.20 | 0.20 |
| GLM | iqr | 0.01 | 0.05 | 0.01 | 0.05 | 0.01 | 0.05 | 0.01 | 0.04 | 0.01 | 0.05 | 0.02 | 0.06 | 0.01 | 0.06 | 0.02 | 0.10 | 0.01 | 0.02 |
| Lasso | median | 0.75 | 0.76 | 0.69 | 0.70 | 0.69 | 0.69 | 0.70 | 0.71 | 0.64 | 0.64 | 0.77 | 0.78 | 0.79 | 0.81 | 0.61 | 0.62 | 0.20 | 0.20 |
| Lasso | iqr | 0.01 | 0.05 | 0.01 | 0.05 | 0.01 | 0.05 | 0.01 | 0.04 | 0.01 | 0.05 | 0.02 | 0.06 | 0.01 | 0.06 | 0.02 | 0.10 | 0.01 | 0.02 |
| ElasticNet | median | 0.75 | 0.75 | 0.69 | 0.69 | 0.69 | 0.69 | 0.70 | 0.71 | 0.64 | 0.63 | 0.76 | 0.78 | 0.78 | 0.79 | 0.61 | 0.60 | 0.24 | 0.23 |
| ElasticNet | iqr | 0.02 | 0.07 | 0.02 | 0.06 | 0.02 | 0.07 | 0.01 | 0.05 | 0.02 | 0.07 | 0.02 | 0.07 | 0.02 | 0.08 | 0.03 | 0.11 | 0.05 | 0.05 |
| Tree boosting | median | 0.76 | 0.76 | 0.72 | 0.72 | 0.72 | 0.72 | 0.72 | 0.72 | 0.69 | 0.69 | 0.76 | 0.78 | 0.75 | 0.75 | 0.70 | 0.71 | 0.19 | 0.19 |
| Tree boosting | iqr | 0.01 | 0.06 | 0.02 | 0.06 | 0.01 | 0.06 | 0.02 | 0.06 | 0.01 | 0.07 | 0.02 | 0.05 | 0.03 | 0.08 | 0.01 | 0.10 | 0.00 | 0.03 |
| MLP | median | 0.75 | 0.76 | 0.71 | 0.71 | 0.71 | 0.70 | 0.71 | 0.70 | 0.68 | 0.67 | 0.76 | 0.75 | 0.76 | 0.75 | 0.70 | 0.75 | 0.21 | 0.23 |
| MLP | iqr | 0.01 | 0.06 | 0.15 | 0.19 | 0.56 | 0.60 | 0.57 | 0.58 | 0.53 | 0.57 | 0.20 | 0.22 | 0.63 | 0.67 | 0.28 | 0.30 | 0.05 | 0.05 |
| SVMC | median | 0.75 | 0.76 | 0.69 | 0.69 | 0.68 | 0.69 | 0.70 | 0.71 | 0.64 | 0.64 | 0.77 | 0.78 | 0.79 | 0.81 | 0.61 | 0.62 | 0.20 | 0.20 |
| SVMC | iqr | 0.01 | 0.05 | 0.02 | 0.06 | 0.02 | 0.06 | 0.01 | 0.06 | 0.02 | 0.06 | 0.02 | 0.07 | 0.01 | 0.08 | 0.03 | 0.12 | 0.01 | 0.02 |
| NB | median | 0.75 | 0.76 | 0.70 | 0.71 | 0.70 | 0.70 | 0.71 | 0.72 | 0.65 | 0.65 | 0.77 | 0.78 | 0.78 | 0.81 | 0.63 | 0.64 | 0.20 | 0.20 |
| NB | iqr | 0.01 | 0.05 | 0.02 | 0.06 | 0.02 | 0.07 | 0.01 | 0.05 | 0.02 | 0.08 | 0.01 | 0.05 | 0.02 | 0.06 | 0.03 | 0.13 | 0.01 | 0.03 |

Feature selection 8 combined features

| **Model** | **Value** | **AUC (training)** | **AUC (test)** | **accuracy (training)** | **accuracy (test)** | **average class accuracy (training)** | **average class accuracy (test)** | **f1 (training)** | **f1 (test)** | **precision PPV (training)** | **precision PPV (test)** | **NPV (training)** | **NPV (test)** | **recall sensitivity (training)** | **recall sensitivity (test)** | **specificity (training)** | **specificity (test)** | **brier score loss (training)** | **brier score loss (test)** |
| --- | --- | --- | --- | --- | --- | --- | --- | --- | --- | --- | --- | --- | --- | --- | --- | --- | --- | --- | --- |
| GLM | median | 0.79 | 0.77 | 0.74 | 0.73 | 0.74 | 0.73 | 0.73 | 0.72 | 0.71 | 0.71 | 0.77 | 0.76 | 0.74 | 0.72 | 0.73 | 0.74 | 0.19 | 0.19 |
| GLM | iqr | 0.02 | 0.06 | 0.02 | 0.07 | 0.02 | 0.07 | 0.02 | 0.06 | 0.02 | 0.07 | 0.02 | 0.07 | 0.02 | 0.11 | 0.02 | 0.07 | 0.01 | 0.03 |
| Lasso | median | 0.78 | 0.77 | 0.73 | 0.72 | 0.73 | 0.72 | 0.72 | 0.72 | 0.69 | 0.69 | 0.77 | 0.77 | 0.76 | 0.75 | 0.71 | 0.71 | 0.19 | 0.19 |
| Lasso | iqr | 0.02 | 0.06 | 0.02 | 0.05 | 0.02 | 0.06 | 0.01 | 0.06 | 0.03 | 0.05 | 0.02 | 0.07 | 0.03 | 0.10 | 0.05 | 0.10 | 0.01 | 0.03 |
| ElasticNet | median | 0.77 | 0.77 | 0.73 | 0.73 | 0.73 | 0.72 | 0.72 | 0.71 | 0.70 | 0.71 | 0.75 | 0.75 | 0.73 | 0.72 | 0.73 | 0.74 | 0.20 | 0.21 |
| ElasticNet | iqr | 0.02 | 0.07 | 0.02 | 0.06 | 0.03 | 0.06 | 0.02 | 0.07 | 0.05 | 0.07 | 0.03 | 0.07 | 0.08 | 0.11 | 0.10 | 0.10 | 0.03 | 0.03 |
| Tree boosting | median | 0.82 | 0.78 | 0.75 | 0.72 | 0.75 | 0.72 | 0.74 | 0.71 | 0.72 | 0.70 | 0.77 | 0.76 | 0.75 | 0.72 | 0.74 | 0.74 | 0.18 | 0.19 |
| Tree boosting | iqr | 0.03 | 0.07 | 0.02 | 0.05 | 0.02 | 0.06 | 0.02 | 0.07 | 0.03 | 0.06 | 0.02 | 0.07 | 0.03 | 0.11 | 0.05 | 0.09 | 0.01 | 0.03 |
| MLP | median | 0.78 | 0.77 | 0.74 | 0.72 | 0.74 | 0.72 | 0.73 | 0.70 | 0.72 | 0.71 | 0.76 | 0.74 | 0.73 | 0.69 | 0.76 | 0.76 | 0.19 | 0.20 |
| MLP | iqr | 0.02 | 0.06 | 0.03 | 0.05 | 0.03 | 0.07 | 0.03 | 0.09 | 0.03 | 0.07 | 0.03 | 0.08 | 0.07 | 0.16 | 0.05 | 0.07 | 0.01 | 0.04 |
| SVMC | median | 0.78 | 0.77 | 0.70 | 0.70 | 0.70 | 0.69 | 0.71 | 0.71 | 0.65 | 0.65 | 0.77 | 0.78 | 0.79 | 0.78 | 0.64 | 0.67 | 0.19 | 0.19 |
| SVMC | iqr | 0.02 | 0.06 | 0.02 | 0.07 | 0.02 | 0.08 | 0.01 | 0.06 | 0.02 | 0.07 | 0.02 | 0.06 | 0.03 | 0.06 | 0.03 | 0.07 | 0.01 | 0.03 |
| NB | median | 0.76 | 0.75 | 0.70 | 0.71 | 0.70 | 0.71 | 0.71 | 0.71 | 0.65 | 0.65 | 0.77 | 0.76 | 0.78 | 0.75 | 0.63 | 0.64 | 0.24 | 0.23 |
| NB | iqr | 0.01 | 0.07 | 0.02 | 0.05 | 0.02 | 0.05 | 0.01 | 0.05 | 0.02 | 0.06 | 0.01 | 0.06 | 0.02 | 0.08 | 0.03 | 0.07 | 0.01 | 0.05 |
